# Long‐Term Complete Remission of Metastatic Endometrial Carcinosarcoma With Radical Surgical Resection and Postoperative Chemotherapy Utilizing Paclitaxel and Carboplatin

**DOI:** 10.1002/cnr2.70346

**Published:** 2025-09-09

**Authors:** Kambiz Novin, Soroush Shahrokh, Mohadese Shahin, Farzad Taghizadeh‐Hesary

**Affiliations:** ^1^ Radiation Oncology Department, School of Medicine Iran University of Medical Sciences Tehran Iran; ^2^ Texas Tech University Health Sciences Center Lubbock Texas USA; ^3^ ENT and Head and Neck Research Center and Department, the Five Senses Health Institute, School of Medicine Iran University of Medical Sciences Tehran Iran

**Keywords:** endometrial carcinosarcoma, long‐term remission, postoperative chemotherapy

## Abstract

**Objective:**

To present a case of metastatic endometrial carcinosarcoma (ECS) with a long‐term complete response to chemotherapy using a paclitaxel and carboplatin regimen.

**Case Report:**

A 47‐year‐old premenopausal woman was diagnosed with a large, advanced intrauterine tumor. She underwent a total abdominal hysterectomy with bilateral salpingo‐oophorectomy. Pathological assessment revealed a uterine mass with mesenchymal and epithelial components consistent with ECS, while the right ovarian tumor was identified as serous papillary carcinoma. The patient experienced a prolonged postoperative recovery period lasting 3 months. Staging workup later revealed recurrence of adnexal and pulmonary lesions, indicating metastasis. The patient received 6 cycles of postoperative chemotherapy consisting of paclitaxel and carboplatin, achieving complete remission that has persisted for 3 years since the initial diagnosis.

**Conclusions:**

This case highlights the efficacy of postoperative chemotherapy using paclitaxel and carboplatin in the successful treatment of metastatic ECS.

## 1 Introduction

Endometrial carcinosarcoma (ECS), also known as malignant mixed Müllerian tumors, is a rare, biphasic aggressive malignant tumor characterized by high‐grade epithelial and mesenchymal neoplastic cell components. Unlike endometrial carcinomas, ECS is classified as a high‐grade tumor by default upon diagnosis. Additionally, ECS is highly prone to early systemic metastasis at initial presentation [1]. As such, while ECS accounts for less than 4% of uterine malignancies worldwide, it is responsible for more than 16% of all uterine cancer‐related deaths each year [2]. Despite advances in uterine cancer treatments and significant improvements in overall uterine cancer survival rates in recent years, ECS continues to pose a significant therapeutic challenge and portends a poor prognosis. This adverse clinical course is further complicated by the challenging diagnosis, the lack of clear treatment guidelines, and the high rate of disease recurrence in ECS [2].

Over 60% of patients with ECS present with advanced disease, and nearly half experience recurrence despite treatment [3]. For patients with advanced‐stage or recurrent ECS, systemic chemotherapy remains the primary treatment. However, the optimal chemotherapeutic regimen continues to be a subject of debate [4]. Here, we present a case of a middle‐aged woman with metastatic ECS who achieved a complete response and prolonged remission following chemotherapy after primary site surgery. Additionally, the relevant literature is reviewed. This case report is unique due to the long‐term remission of a patient with metastatic ECS. The treatment protocol used can inform the design of future clinical trials.

## 2 Case Presentation

A 47‐year‐old premenopausal woman (G1P1L1) with a history of type 2 diabetes mellitus and deep venous thrombosis (DVT) presented with progressively worsening vaginal bleeding. The patient's medical records did not provide specific information on the cause, treatment, or timing of the DVT. She was in a monogamous relationship with her husband and had no history of sexually transmitted diseases, familial cancer syndromes, alcohol consumption, or tobacco use. The patient initially attributed her irregular vaginal bleeding to menopause but sought medical attention as symptoms worsened. She was first seen in January 2021 at the Radiation Oncology Department of Shohadaye Haft‐e‐Tir Hospital (Tehran, Iran). The patient's management involved a multidisciplinary team, including radiation oncologists, surgeons, radiologists, and pathologists, ensuring a comprehensive treatment approach.

## 3 Investigations

**FIGURE 1:**
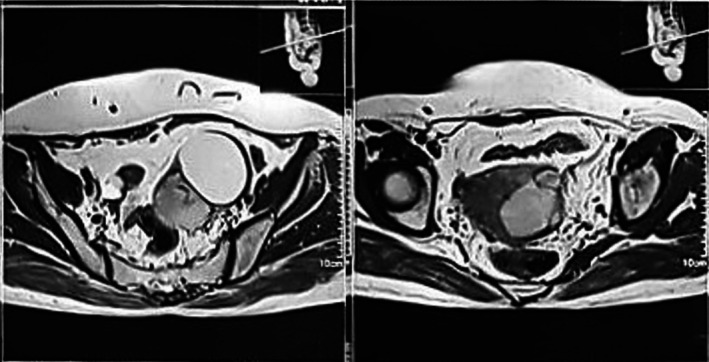
Patient's presurgical MRI of the abdomen and pelvis revealed a 7.4 × 7.3 × 5.7 cm cystic and solid lesion in the left adnexa extending into the left ovary and uterine wall, and a 3.6 × 3.3 × 1.7 cm cystic and hemorrhagic lesion in the right adnexa extending into the right ovary and the right uterine wall.

The patient underwent an abdominopelvic ultrasound, which revealed a solid and cystic lesion in the left adnexa measuring 9.6 × 6.8 × 6.5 cm in diameter. Laboratory studies showed normal levels of β‐human chorionic gonadotropin (β‐hCG), lactate dehydrogenase (LDH), α‐fetoprotein (AFP), and CA‐19‐9. However, the CA‐125 level was elevated at 128 U/mL (normal < 35 U/mL). Abdominopelvic magnetic resonance imaging (MRI) revealed a 7.4 × 7.3 × 5.7 cm cystic and solid lesion in the left adnexa extending into the left ovary and uterine wall, and a 3.6 × 3.3 × 1.7 cm cystic and hemorrhagic lesion in the right adnexa extending into the right ovary and right uterine wall (Figure 1).

The patient underwent a diagnostic laparotomy and total abdominal hysterectomy with bilateral salpingo‐oophorectomy (TAH‐BSO). Intraoperatively, a uterine mass with invasion into the rectal wall was found, forming an adhesion between the outer uterine wall and the rectum. Additionally, a polypoid lesion was found originating from the outer uterine wall extending to the intestinal wall. Further resection was performed to remove the uterine mass adherent to the rectum, the polypoid lesion extending from the uterine wall to the intestinal wall, and the omentum. However, no resection‐anastomosis procedure was necessary, as the lesion was removed without requiring intestinal resection or reconnection. The resected tissues were then sent for pathological evaluation.

**FIGURE 2:**
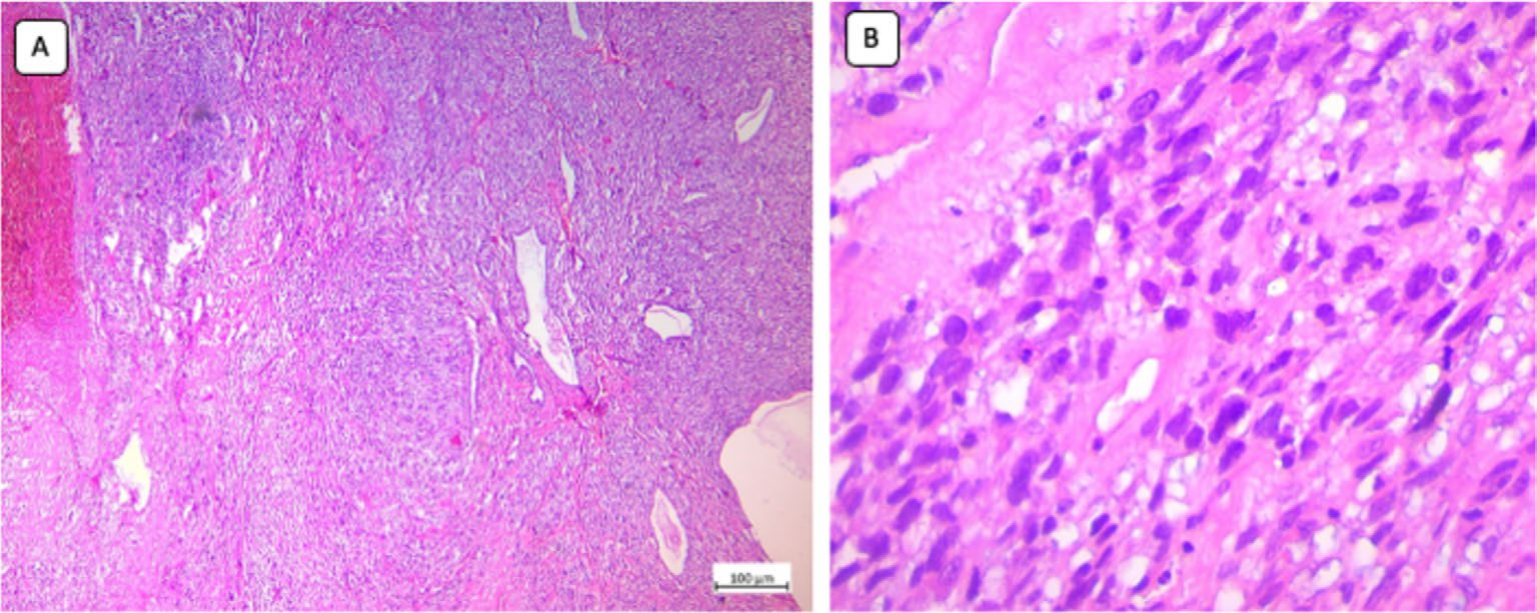
(A) Microscopic examination of the left ovarian lesion revealed a biphasic lesion with malignant proliferation of spindle and oval‐shaped cells in a diffuse fascicular pattern with focal areas of necrosis (×40). (B) Neoplastic cells were characterized by pleiomorphic, hyperchromatic nuclei with cellular atypia, along with foci of gland formation by neoplastic epithelial cells (×100).

Microscopic examination of the left ovarian tumor revealed a biphasic lesion with malignant proliferation of spindle and oval‐shaped cells in a diffuse fascicular pattern accompanied by focal areas of necrosis. The neoplastic cells exhibited pleomorphic, hyperchromatic nuclei with cellular atypia, along with foci of glandular formation by neoplastic epithelial cells (Figure 2). Further evaluation with immunohistochemistry of the lesion revealed positive immunostaining for β‐catenin, CD10, and vimentin and negative immunostaining for LCA, inhibin, desmin, estrogen receptor, and cytokeratin tumor markers, consistent with high‐grade carcinoma.

The microscopic examination of the uterus, right ovary, and rectal wall revealed carcinosarcoma originating from the uterine endometrium with extension into the uterine serosa, medial aspect of the right ovary, intestinal and rectal wall, and invasion of the lymphovascular system. Clinical staging of uterine carcinosarcoma was PT4NxMx (based on AJCC/FIGO). The microscopic examination of the lateral right ovarian mass revealed serous papillary carcinoma, while the omentum was negative for peritoneal seeding and the cervix showed chronic cervicitis. The cytological analysis of the peritoneal fluid only revealed reactive mesothelial cells but no evidence of metastasis. Although the patient had ovarian serous carcinoma, lymphadenectomy was not performed. The primary focus of the surgery was on resecting the uterine carcinosarcoma with its advanced local invasion. Given the clinical staging of T4 for the uterine carcinosarcoma and the absence of clinical or radiological evidence of lymph node involvement, the surgical team opted not to perform lymphadenectomy. Postoperative serum tumor markers revealed normalized CA‐125 (26.2 U/mL) and CEA (2.6 ng/mL) (normal range CEA: 0–2.5 ng/mL).

**FIGURE 3:**
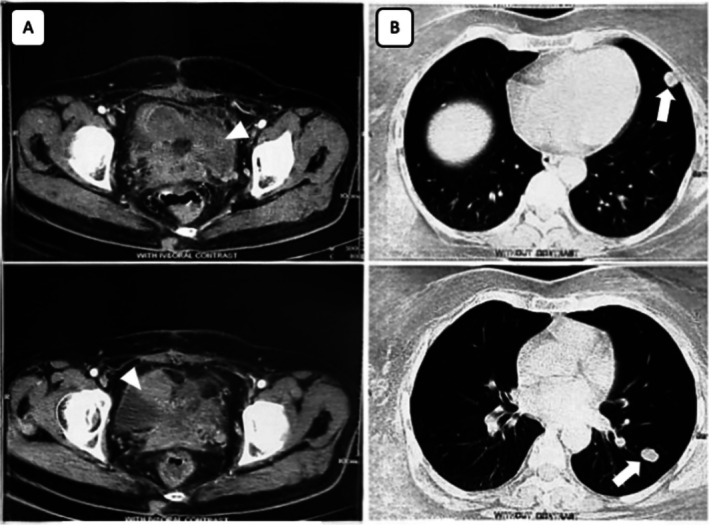
(A) Patient's postoperation CT of abdomen and pelvis revealed two large heterogeneous hyper‐enhancing adnexal lesions in the pelvic cavity in place of each ovary, measuring 5.5 × 8.3 cm on the right and 6.4 × 5.1 cm on the left (arrow heads). (B) CT of the chest revealed multiple nodular, hyper‐enhancing noncalcified lymphadenopathy measuring greater than 1.0 cm in size in various regions of the lungs and mediastinum, consistent with metastatic disease (arrows).

The patient had a prolonged postoperative recovery period lasting 3 months. Approximately 3 months after the operation, she was referred to our oncology clinic for postoperative chemotherapy. Abdominopelvic computed tomography (CT) scan revealed two large heterogeneous hyper‐enhancing cystic lesions in bilateral adnexa in place of each ovary with invasion into a long segment of the sigmoid colon (Figure 3A). Chest CT revealed multiple nodular, hyper‐enhancing noncalcified enlarged lymph nodes in multiple regions of the lungs and mediastinum, primarily consistent with metastatic lymphadenopathy (Figure 3B). Serum tumor markers revealed reduced CA‐125 (5.6 U/mL) and CEA (2.6 ng/mL). Although a biopsy of the new lung lesions might have provided additional information on the metastatic origin, the typical radiographic features, combined with the advanced stage of the primary uterine carcinosarcoma, led the treating physician to proceed with chemotherapy without biopsy confirmation.

## 4 Treatment

**FIGURE 4:**
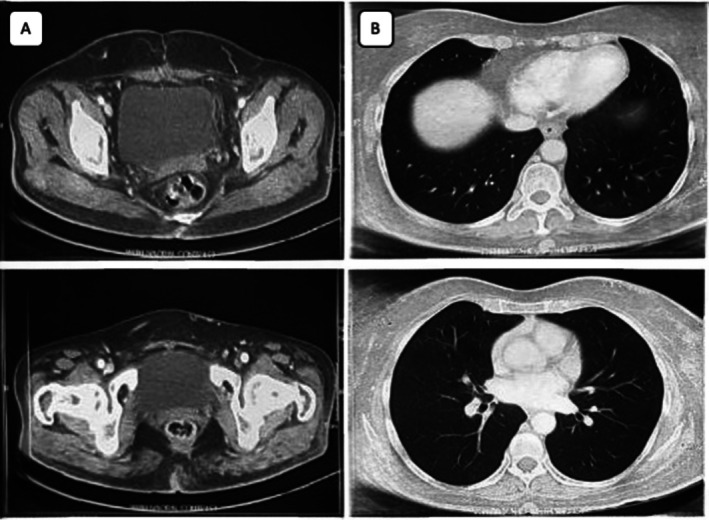
CT of the chest, abdomen, and pelvis obtained after the completion of the fourth cycles of chemotherapy revealed eradication of the lung and mediastinal lymphadenopathy, along with a significant decrease in the size of the pelvic and ovarian lesions.

The patient commenced postoperative chemotherapy with carboplatin and paclitaxel. Initially, she received carboplatin 350 mg and paclitaxel 230 mg, with subsequent increments to carboplatin 420 mg and paclitaxel 260 mg over the next 4 cycles. A CT scan revealed complete resolution of lung and mediastinal lymphadenopathy, along with a significant reduction in the size of pelvic and ovarian lesions (Figure 4). However, her chemotherapy regimen was complicated by the onset of severe distal neuropathy in her bilateral upper and lower extremities following the fourth cycle, which was attributed to a paclitaxel‐related adverse reaction. As a result, the paclitaxel dosage was reduced from 260 to 240 mg per cycle, while the carboplatin dosage was increased from 420 to 450 mg per cycle.

## 5 Outcome and Follow‐Up

After 6 cycles of chemotherapy, the patient experienced no further adverse effects. Follow‐up CT scans confirmed ongoing remission (Figure 4) with subsequent scans at 6, 12, 24, and 36 months showing no signs of recurrence. Written informed consent was obtained from the patient for the publication of this case report and any accompanying images.

## 6 Discussion

ECS, a rare uterine neoplasm with carcinomatous and sarcomatous components, is highly aggressive [1]. More than 60% of ECS patients present with advanced disease, and nearly half experience recurrence even after treatment. Diagnostic challenges, unclear pathogenesis, and limited chemotherapy options contribute to a poor prognosis, with a 0%–10% five‐year survival rate for stage III–IV disease [2]. Risk factors for ECS align closely with those of endometrial carcinoma, including hyperestrogenism, obesity, diabetes, nulliparity, tamoxifen use, and pelvic radiation exposure [3]. In addition, breast cancer survivors undergoing hormonal treatment are at greater risk of endometrial malignancies [5]. While ECS typically affects older women, our patient, aside from diabetes and obesity, lacked common predisposing factors, highlighting the need to consider ECS in premenopausal women with relevant symptoms [6].

ECS, once considered a primary uterine sarcoma, is now classified as metaplastic carcinoma. While the exact cause of ECS remains unclear, proposed pathogenesis theories include collision, combination, composition, and conversion hypotheses, offering varied insights into the origin of this aggressive tumor [7]. The collision, combination, and composition theories were commonly accepted in the past. Recent advances in molecular and cytogenetic analysis have revealed a close resemblance of the genomic aberrations between the various cell types in ECS, including mutations in KRAS, TP53, c‐Myc, PIK3CA, and PTEN [7].

Diagnosing ECS is challenging due to its sporadic presentation and lack of specific clinical or imaging features. While imaging is nonpathognomonic, it aids in the initial diagnosis, staging, and posttreatment monitoring. CT scans typically reveal a heterogeneous, ill‐defined, hypodense lesion with internal hemorrhage and necrosis, and this limitation extends to MRI as well. A recent study by Li et al. reported that MRI has an accuracy of just 3.3% in detecting ECS [8]. In retrospect, the MRI report in our case did not clearly identify the primary origin of the uterine carcinosarcoma, likely due to two key factors: (1) the tumor's complex, biphasic structure, which may have been difficult to distinguish as a discrete mass and (2) the MRI's emphasis on the adnexal lesions and their extension into the uterine wall, which may have obscured the primary uterine mass. While MRI remains a valuable tool for evaluating adnexal involvement and staging, its limitations in detecting complex, biphasic tumors such as ECS underscore the need for additional diagnostic approaches, including biopsy, to ensure accurate diagnosis and treatment planning.

The only significant tumor marker in ECS is CA‐125, which may be used for initial diagnosis, monitoring treatment response, and screening for disease recurrence. Other serum tumor markers, such as AFP, β‐hCG, LDH, CA 15‐3, CA 19‐9, and CEA, have no diagnostic value in ECS [9]. These findings were consistent with serum tumor marker analysis and findings in our patient. Upon initial presentation, she was found to have normal levels of AFP, CA 19‐9, CEA, and β‐hCG, while her CA‐125 was elevated. After undergoing TAH‐BSO, the patient had a marked decrease in CA‐125, bringing it within the normal range. Interestingly, however, her subsequent measurements of CA‐125 after initiation of adjuvant chemotherapy revealed a consistent decrease. This highlights the value of CA‐125 in diagnosing ECS. Furthermore, it suggests that, despite reaching the normal range postoperative, trends in serum CA‐125 levels may be useful in assessing a patient's treatment response.

Treatment of ECS depends on the patient's functional status and disease stage. For patients with local tumors or metastatic disease confined to the peritoneal cavity, surgical resection with TAH‐BSO, omentectomy, and pelvic and para‐aortic lymphadenectomy is the mainstay treatment [3]. The role of adjuvant chemotherapy remains unclear because of the insufficient data on the disease. A Cochrane review reported increased overall survival in patients with ECS stage IB or higher who received adjuvant chemotherapy [10]. Meanwhile, in patients with advanced‐stage or recurrent ECS, the primary modality of treatment is systemic chemotherapy [11]. Although the optimal chemotherapeutic regimen is still debated, the best survival outcomes and reduction in disease progression were observed with combination chemotherapy using ifosfamide and paclitaxel [12]. Previously, ECS misclassification as mesenchymal tumors led to the use of ifosfamide, replaced by carboplatin due to toxicity. Our patient achieved complete remission with carboplatin and paclitaxel, suggesting that higher carboplatin doses could be considered in patients intolerant to high‐dose paclitaxel. While a biopsy of the metastatic lung lesions would have provided further insights into their exact origin, the decision not to biopsy was based on typical radiographic findings that suggested metastasis from the uterine carcinosarcoma. Given the advanced stage of the primary tumor and the known responsiveness of both the carcinomatous and sarcomatous components to paclitaxel and carboplatin, the treatment strategy was unlikely to change regardless of the biopsy result. Nonetheless, in cases where biopsy is feasible, it could potentially offer more precise information for guiding management and prognosis.

This case report has several limitations. First, the metastatic lesions identified in the lungs were not biopsied; thus, their histological origin—whether from uterine carcinosarcoma or serous ovarian carcinoma—could not be definitively confirmed. Second, lymphadenectomy was not performed due to intraoperative complexity, prolonged surgical time, and the absence of evident nodal involvement on imaging or palpation, limiting accurate nodal staging. Lastly, although MRI identified bilateral adnexal masses, it failed to detect the full extent of uterine and rectal wall invasion. This may be attributed to the biphasic morphology of the tumor and technical limitations, underscoring the need for careful correlation with clinical and surgical findings in such complex cases.

## 7 Conclusions

There is no standard treatment for patients with advanced ECS. This case report underscores the efficacy of using paclitaxel and carboplatin as a postoperative chemotherapy regimen in the successful management of metastatic ECS. These findings provide valuable insights for future research to enhance survival of metastatic ECS.

## Author Contributions

**M.S.:** conceptualization, writing – original draft, validation, supervision. **K.N.:** visualization, investigation. **S.S.:** writing – original draft. **F.T.H.:** supervision, writing – review and editing.

## Conflicts of Interest

The authors declare no conflicts of interest.

## Data Availability

The data that support the findings of this study are available from the corresponding author upon reasonable request.

## References

[1] S. A. El‐Nashar and A. Mariani , “Uterine Carcinosarcoma,” Clinical Obstetrics and Gynecology 54, no. 2 (2011): 292–304, 10.1097/GRF.0b013e31821ac635. 21508698

[2] N. Nama , F. D. Cason , S. Misra , et al., “Carcinosarcoma of the Uterus: A Study From the Surveillance Epidemiology and End Result (SEER) Database,” Cureus 12, no. 9 (2020): e10283, 10.7759/cureus.10283. 33042718 PMC7538206

[3] G. Bogani , I. Ray‐Coquard , N. Concin , et al., “Endometrial Carcinosarcoma,” International Journal of Gynecological Cancer 33, no. 2 (2023): 147–174, 10.1136/ijgc-2022-004073. 36585027

[4] D. Lorusso , “What Is the Best Chemotherapy Regimen for Uterine Carcinosarcoma?: Carboplatin/Paclitaxel,” in 50 Big Debates in Gynecologic Oncology, ed. D. S. Chi , N. Lakhi , and N. Colombo (Cambridge University Press, 2023), 229–231, 10.1017/9781108935579.075.

[5] S. G. Vitale , S. Angioni , M. N. D’Alterio , et al., “Risk of Endometrial Malignancy in Women Treated for Breast Cancer: The BLUSH Prediction Model—Evidence From a Comprehensive Multicentric Retrospective Cohort Study,” Climacteric 27, no. 5 (2024): 482–488, 10.1080/13697137.2024.2376189. 39023103

[6] A. Gadducci , S. Cosio , A. Romanini , and A. R. Genazzani , “The Management of Patients With Uterine Sarcoma: A Debated Clinical Challenge,” Critical Reviews in Oncology/Hematology 65, no. 2 (2008): 129–142. 17706430 10.1016/j.critrevonc.2007.06.011

[7] O. Gotoh , Y. Sugiyama , Y. Takazawa , et al., “Clinically Relevant Molecular Subtypes and Genomic Alteration‐Independent Differentiation in Gynecologic Carcinosarcoma,” Nature Communications 10, no. 1 (2019): 4965, 10.1038/s41467-019-12985-x. PMC6823358 31672974

[8] L. Li , W. Huang , K. Xue , et al., “Clinical and Imaging Features of Carcinosarcoma of the Uterus and Cervix,” Insights Into Imaging 12, no. 1 (2021): 142, 10.1186/s13244-021-01084-5. 34674042 PMC8531181

[9] N. Thomakos , A. Rodolakis , F. Zagouri , et al., “Serum CA 125, CA 15‐3, CEA, and CA 19‐9: A Prognostic Factor for Uterine Carcinosarcomas?,” Archives of Gynecology and Obstetrics 287, no. 1 (2013): 97–102, 10.1007/s00404-012-2529-6. 22941327

[10] K. Galaal , K. Godfrey , R. Naik , A. Kucukmetin , and A. Bryant , “Adjuvant Radiotherapy and/or Chemotherapy After Surgery for Uterine Carcinosarcoma,” Cochrane Database of Systematic Reviews 1 (2011): CD006812. 10.1002/14651858.CD006812.pub2 PMC4161119 21249682

[11] J. McEachron , T. Heyman , L. Shanahan , et al., “Multimodality Adjuvant Therapy and Survival Outcomes in Stage I–IV Uterine Carcinosarcoma,” International Journal of Gynecological Cancer 30, no. 7 (2020): 1012–1017, 10.1136/ijgc-2020-001315. 32447295

[12] H. D. Homesley , V. Filiaci , M. Markman , et al., “Phase III Trial of Ifosfamide With or Without Paclitaxel in Advanced Uterine Carcinosarcoma: A Gynecologic Oncology Group Study,” Journal of Clinical Oncology 25, no. 5 (2007): 526–531, 10.1200/JCO.2006.06.4907. 17290061

